# Contributions of multiple refugia during the last glacial period to current mainland populations of Korean pine (*Pinus koraiensis*)

**DOI:** 10.1038/srep18608

**Published:** 2015-12-22

**Authors:** Lei Bao, Ayijiamali Kudureti, Weining Bai, Rongzhang Chen, Tianming Wang, Hongfang Wang, Jianping Ge

**Affiliations:** 1 State Key Laboratory of Earth Surface Processes and Resource Ecology, Ministry of Education Key Laboratory for Biodiversity Science and Engineering & College of Life Sciences, Beijing Normal University, Beijing, China

## Abstract

The northern microrefugia that existed during the Last Glacial Maximum (LGM) are a key factor in the demographic history of species. *Pinus koraiensis* has a unique distribution in northeast Asia. The Changbai Mountains and the Korean peninsula (CM/KP) are usually considered to be the LGM refugia for *P. koraiensis*. However, the Xiaoxingan Range (XR), at the northern part of this species’ distribution, is another possible refugium. We used chloroplast sequencing and ten nuclear single-copy gene loci to calculate the genetic diversity pattern of *P. koraiensis*. The probabilities of a single LGM refugium and of multiple LGM refugia were calculated based on approximate Bayesian computation. The effect of the latitudinal gradient on genetic diversity was not significant. However, unique alleles occurred at low frequencies in CM/KP and XR. A conservative estimate of the coalescence time between CM/KP and XR is 0.4 million years ago, a time prior to the LGM. Gene flow between CM/KP and XR was estimated to be more than one in per generation, an amount that may be sufficient to limit genetic divergence between the regions. Our study strongly supports the hypothesis that XR was another LGM refugium in addition to CM/KP.

Historical climate change, especially during the last glacial maximum(LGM, ca. 18,000–24,000 years before the present), has affected the demographic history of species and shaped modern distributions. Enlarged ice caps in North America and Europe during the LGM caused significant local extinctions of temperate flora in their northern ranges^1^,^2^. In East Asia, extensive land ice caps did not develop^3^, but pollen record data show that reduced temperatures (mean reduction =7–10 °C) and increased aridity caused extensive local extinctions (>30 °N)^4^,^5^,^6^. For plants currently restricted to high latitudes, it is typically unknown which of their northern populations went extinct during the LGM. Did some populations survive in Northern microrefugia? The discovery and analysis of northern LGM microrefugia is important for better understanding evolution and range shifts under global climate change^7^,^8^.

Korean pine (*Pinus koraiensis* Siebold & Zuccarini) is distributed in Northeast China and adjacent regions including the Korean peninsula and the Russian Far East (hereafter, NEA) and scattered locations in the Japanese archipelago. Pollen records indicate that mixed forest in NEA was replaced by boreal forest, tundra or steppe during the LGM. Very low amounts of *Pinus* pollen have been found during this period^4^,^9^,^10^, which may due to rare locations with LGM pollen records in this region. Korean pine populations may have declined or disappeared in most of the modern range of this species. Which location(s) were possible refugia for Korean pine population survival during the cold LGM period? Mitochondrial DNA markers indicate clear genetic differentiation between the Japanese archipelago and the NEA populations, suggesting that the Japanese archipelago was a refugium independent from the Asian continent during the LGM^11^. However, because very low genetic diversity has been found in mitochondrial and chloroplast DNA markers, the possible location(s) for NEA population survival during the LGM are unknown.

The Changbai Mountain Range, located in southern NEA, has the only mountains in this region with altitudes >2000 m (maximum, 2749 m). The Changbai Mountains (CM) can act as a barrier blocking cold air masses from Siberia. Therefore, the southern CM and the Korean peninsula (KP) are areas that potentially supported relict populations of Korean pine during the LGM. Existing phylogeographic studies on plants distributed in NEA have found relatively higher genetic diversity in CM and/or KP, suggesting possible LGM refugia in CM and/or KP^12^. Examples of tree species represented by this pattern include *Juglans mandshurica*^13^, *Quercus mongolica*^14^, *Fraxinus mandschurica*^15^, and *Acer mono*^16^. It has been suggested that Korean pine had LGM refugia in CM and/or KP. Based on allozyme and RAPD markers, high genetic diversity was found in Korean pine populations in CM and KP^17^,^18^,^19^. Relatively higher concentrations of *Pinus* pollen in CM may also support the CM/KP refugia hypothesis^9^. Because no modern Korean pine populations occur outside of NEA, it is believed that CM/KP is a Korean pine refugium^11^,^20^. Continuing debate on LGM refugia of Korean pine has focused on the numbers of LGM refugia^11^,^19^,^21^ and the search for additional refugia north of CM, such as in the Xiaoxingan Range (XR, Fig. 1).

Kremenetski *et al.*^21^ suggested that the limiting factor for Korean pine survival during the LGM was not simply low temperatures but also aridity. According to this hypothesis, XR might have served as another LGM refugium because it was more humid. Thus far, neither genetic studies nor paleovegetation reconstruction has provided support for the multiple refugia hypothesis. Inconsistent genetic patterns of Korean pine populations have been reported. Significant genetic clines were found by Kim *et al.*^17^ using allozyme markers, suggesting a single refugium in CM/KP. Another allozyme study found no genetic clines with populations in the Russian Far East (north of XR) with high genetic diversity equal to CM populations^19^. These studies were conducted >10 years ago and were limited by available molecular markers and older phylogeographic statistics. In a more recent study, using mitochondrial and chloroplast DNA markers, Aizawa *et al.*^11^ found very low genetic diversity in NEA and suggested CM/KP as the LGM refugia for the Asian continental populations. However, because some populations north of CM have distinctive chloroplast haplotypes, the possibility of another LGM refugium in the northern region cannot be excluded. The range of Korean pine in Asia is restricted to NEA. Hence, low genetic diversity in plasmids may limit the ability to recover the demographic history of this species in Asia. Abundant nuclear single-copy DNA (nsc DNA) markers are now available for the genus *Pinus*^22^,^23^,^24^,^25^, providing an opportunity to more thoroughly assess the possible location(s) of the refugia.

In this study, we utilized multiple nsc DNA markers and robust statistical phylogeographical approaches. Our goal was to assess the possibility of XR as another LGM refugium. The specific questions asked are as follows: (1) Do NEA populations have a genetic diversity gradient related to latitude? Does the XR population have unique alleles? (2) What were the coalescence times of the continental Asian Korean pine populations before and after the LGM? (3) What was the direction of migration between the CM and the XR populations? If only one LGM refugium existed, in CM/KP, the XR populations probably originated from postglaciation expansion from the south. Hence, we would expect a significant cline in genetic diversity from south to north, and no unique alleles would be expected in XR populations. Based on this hypothesis, the coalescence time of the continental populations would be after the LGM, and a northward migration would be expected. If LGM refugia existed in both CM/KP and XR, a cline in genetic diversity is less likely. Unique alleles might exist in both CM/KP and XR populations. In a multiple refugia hypothesis, we would expect a long coalescence time for the continental populations, at least before the LGM. Both northward and southward migrations are expected if both CM/KP and XR supported relict populations during the LGM.

## Results

### Genetic diversity of each locus

**Figure 1:**
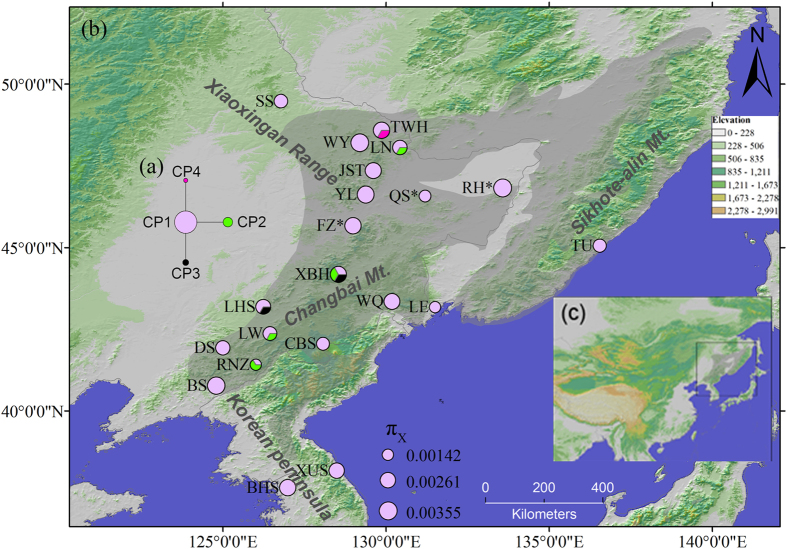
(**a**) Median-joining network of chloroplast haplotypes, calculated in Network 4.6.1.2. (**b**) Chloroplast haplotype distribution over the 21 sampled populations. The colors in the pie chart correspond to the haplotype colors shown in (**a**). The circle size represents the pairwise nucleotide genetic differences within populations (*π*_X_). The three populations (FZ, QS and RH) marked with stars were at the northern border of the Changbai Mts. and were not included in the msABC analysis. (**c**) Map of East Asia. The box indicates our study area. Maps (**b**,**c**) were generated in ArcMap 9.3 (ESRI Inc).

The total length of the chloroplast intergenic fragment *psbA*-*trnH* was 597 bp. This fragment generated four haplotypes in the continental populations, with three polymorphic sites. The grey haplotype, CP1, was most dominant, occurring in all studied populations. The less frequent CP2 haplotype was found in both CM and XR. CP3 was only found in CM (populations LHS and XBH), while CP4 was only found in XR (population TWH) (Fig. 1).

Among the 10 nsc loci we sequenced, the lengths varied from 336 bp to 738 bp, with the exon length varying from 0 bp to 416 bp and the intron length varying from 54 bp to 647 bp (Table 1). Each nsc locus contained 2–19 segregating sites and produced 3–12 haplotypes (Table 2). The average total nucleotide diversity (*π*_t_) was 0.00295, ranging from 0.00866 (*a3ip2*) to 0.00023 (*CL180Contig1_03*). The nucleotide diversity of silent sites (*π*_s_) was 0.00537 (0–0.00866), and that of nonsynonymous sites (*π*_a_) was 0.00062 (0–0.00285). Detailed information on the genetic diversity of each locus is presented in Table 2. According to statistical results based on Tajima’s *D*, Fu and Li’s D* and F*, no nsc loci departed significantly from the neutral hypothesis (Table 2).

**Figure 2:**
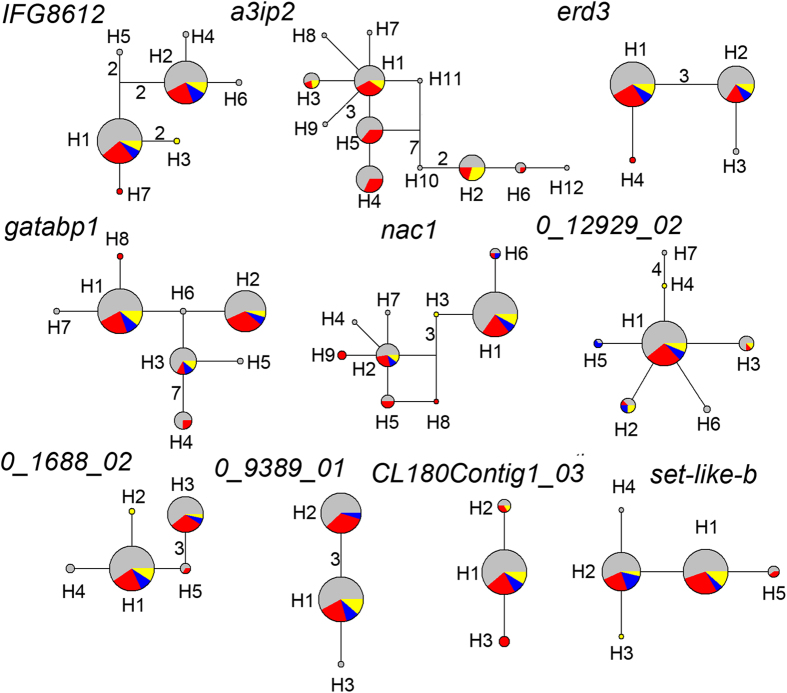
Median-joining network of haplotypes found in each of the ten nuclear single-copy loci. Color scale represents haplotypes found in different regions: grey, Changbai Mts.; red, Xiaoxingan Range; yellow, Korean peninsula; blue, Sikhote-Alin Mts.

Median-joining networks of haplotypes found in every 10 nsc loci indicated that 8 out of 10 loci had two or more dominant haplotypes and a frequency greater than 10%. Loci *0_12929_02* and *CL180Contig1_03* only had one dominant haplotype, with frequencies of 85.8% and 93.2%, respectively. Dominant haplotypes of all 10 nsc loci occurred in all regions, including KP, CM, XR and the Sikhote-Alin Mts. Unique alleles were found in all regions except the Sikhote-Alin Mts. CM regions had unique alleles at all loci except*PK169*, whereas XR had unique alleles at five loci (*IFG8612*, *erd3*, *gatabp1*, *nac*, *PK169*), and KP also had unique alleles at five loci (*IFG8612*, *nac*, *PK20*, *PK47*, *setb*) (Fig. 2).

### Latitudinal gradient of genetic diversity

The genetic diversity of each population (*π*_X_) ranged from 0.0014 to 0.0036. Two CM populations, RNZ and TU, had the lowest genetic diversity (0.0014), while a northern marginal population of CM (RH) had the highest genetic diversity (0.0036). Another two populations, BS (in CM) and YL (in XR), had relatively high genetic diversity (0.0033 and 0.0031, respectively) (Table S1, Fig. 1). After adjusting for the effect of sample size, two CM populations (BS and DS) had the highest allelic richness (*AR*) and private allelic richness (*PAR*), while CBS (in CM) had the lowest *AR* and *PAR* (Table S1).

Pearson correlation analyses indicated no significant correlation between latitude and *π*_X_ (*R* = 0.127, *P* = 0.583), *AR* (*R* = 0.220, *P* = 0.443), or *PAR* (*R* = -0.475 *P* = 0.073). A Mantel test showed no significant pattern of isolation by distance (*R* = 0.110, *P* = 0.160).

### Historical demography analysis

**Figure 3:**
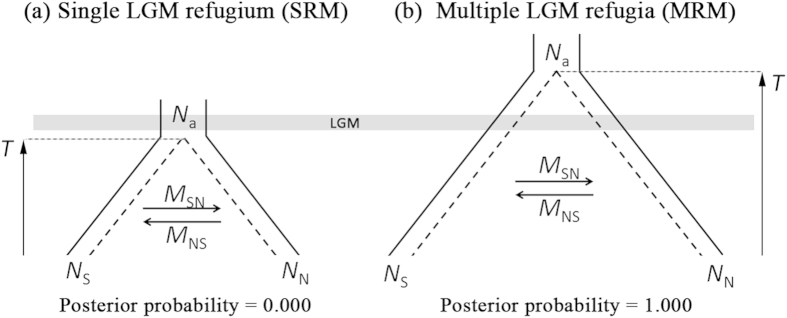
Two demographic models of the coalescence time of two regional Korean pine populations, Changbai Mts. (including adjacent regions) and Xiaoxingan Range. Their corresponding posterior probability estimated with the ABC model selection procedure is shown below the model. (**a**) Single LGM refugium model (SRM): the coalescence time of the two regional populations was previous to the LGM. (**b**) Multiple LGM refugia model (MRM): the coalescence time of the two regional populations was subsequent to the LGM. *N*_N_ and *N*_S_ denote the long-term effective population size of the Changbai Mts. (including the adjacent regions) and Xiaoxingan Range, respectively. *N*_a_ is the effective population size of the most common recent ancestor of the two regional populations. *M*_SN_ and *M*_NS_ are the long-term average gene flow from the Changbai Mts. to the Xiaoxingan Range and from the Xiaoxingan Range to the Changbai Mts., respectively. *T* denotes the coalescence time of the two regional populations.

The genetic diversity pattern of continental Korean pine populations fit the MRM best, with the posterior probability equal to 1 (Fig. 3). The Bayes factor of the MRM and SRM was much larger than 10, and this result strongly supported the MRM scenario. This model selection result meant that the CM/KP populations and the XR populations had a long coalescence time before the LGM and suggested that both the CM/KP and XR populations existed before the LGM.

**Figure 4:**
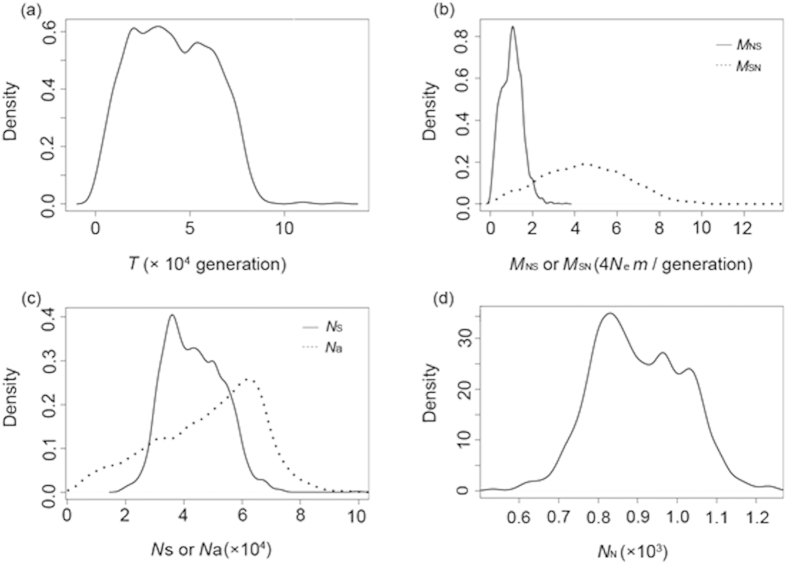
Probability densities of six demographic parameters for the multiple LGM refugia models for Korean pine. (**a**) *T*; (**b**) *M*_SN_(dotted line) and *M*_NS_(solid line); (**c**) *N*_S_(solid line) and *N*_a_(dotted line); (**d**) *N*_N_. The legend is the same as that of Fig. 3.

The long-term effective population size of the CM/KP region was nearly 50 times larger than that of the XR region (median N_S_ = 4.34 × 10^4^ and N_N_ = 0.09 × 10^4^, Table 3, Fig. 4). The long-term migration between CM/KP and XR was large in both directions. The migration from CM/KP to XR per generation (*N*_*e*_*m*) was slightly larger than 1 individual (1.08, 95% HPD: 0.16–2.08), while the contrasting direction of migration was less than 1 individual (0.24, 95% HPD: 0.06–0.55). The coalescence time of the CM/KP and XR populations was 3.96 × 10^4^ generations ago (95% HPD: 0.50 × 10^4^–7.58 × 10^4^). The ancestral population was slightly larger than the population of the CM/KP region (median N_A_ = 5.39 × 10^4^).

## Discussion

Based on ten nsc DNA loci and one chloroplast intergenic fragment, we found low genetic diversity within mainland populations of Korean pine. This result was consistent with another report using mitochondria and chloroplasts by Aizawa *et al.*^11^. We found four chloroplast haplotypes, of which one was dominant and widespread in all populations. The distribution pattern and chloroplast diversity of Korean pine were similar to those of other sympatric trees, such as *Pinus densiflora* (four chlorotypes), *P. sylvestris* var. *mongolica* (six chlorotypes)^25^, *Larix sibirica* (one chlorotype)^26^, *Juglans mandschurica* (one chlorotype)^13^, *Quercus mongolica* (four chlorotypes)^14^, and *Acer mono* (four chlorotypes)^16^. The nucleotide diversities of the ten nsc loci were 0.00295 (*π*_t_), 0.00062 (*π*_a_), and 0.00537(*π*_s_), which were consistent with levels of genetic diversity reviewed by Leffler *et al.*^27^ but lower than the genetic diversity in four sympatric *Pinus* species^25^. For example, among the four pine species studied by Ren *et al.*^25^, *P. sylvestris* var. *mongolica* had the lowest diversity based on seven nsc loci (*π*_t_ = 0.00579, *π*_a_ = 0.00172, *π*_s_ = 0.00759), but this was greater than that of Korean pine. Populations of Korean pine from the CM/KP and XR regions had unique alleles in the chloroplast and nuclear genome, but the latitudinal gradient of population genetic diversity was not significant. ABC simulations strongly supported the theory that CM/KP and XR regions had an ancestral coalescence time long before the LGM. These findings suggest that the continental Korean pine probably had multiple refugia populations during the LGM.

We found no significant gradients related to latitude and no pattern of isolation by distance (P > 0.05). Many populations located in CM/KP had high genetic variation and uniqueness (e.g., population RH, BS and YL, Fig. 1). Both a single refugium and a multiple-refugia scenario could produce such a pattern. Due to possible genetic drift along the leading edge, population expansion would generally be accompanied by a loss of genetic variation during the expansion period^28^. We would therefore expect postglacial expansion from a single refugium located in CM/KP to generate a latitudinal gradient of genetic diversity and isolation by distance pattern with the refugium population containing the highest genetic variation and uniqueness (such as for *F. mandschurica*^15^, *Q. mongolica* (Zeng *et al.* unpublished data), and *J. mandshurica*^13^ in the same region). If postglacial expansion evolved from multiple refugia, genetic variation loss during expansion would not be correlated with latitude and there would not be a clear pattern of isolation by distance. Single or multiple genetic diversity center(s) are both possible in the multiple-refugia scenario. However, a single diversity center in this multiple-refugia scenario would have originated from a mixture of migration events from multiple refugia, and this would reduce the uniqueness of genetic diversity^29^. Based on our results, the diversity pattern of Korean pine on the Asian continent supports the notion that there were refugia in addition to the existing CM/KP area. However, we should be cautious when inferring multiple refugia based on the absence of a latitude gradient and the diversity center locations, because in some situations, single refugium might generate a similar pattern. Pines are usually considered to be species with a high dispersal ability because pollen can, in some instances, travel more than 1000 kilometers^30^,^31^. Hence, for pine species, significant gene flow via pollen might prevent latitude gradients. For example, *Picea chihuahuana*, naturally distributed in Northwestern Mexico, shows two spatially separated mitotypes, which disperse via seeds. In contrast, no phylogeographic structure was found from chloroplast markers, which are dispersed via pollen in conifer species^32^. The distribution of Korean pine on the Asian continent extends less than 2000 kilometers between its southern and northern margins. Therefore, we cannot rule out the possibility of a single refugium if our inferences are based only on patterns of latitude gradient and isolation by distance.

Fortunately, multiple nuclear gene markers and the development of statistical phylogeography can provide more details of the demographic history of Korean pine. ABC simulations suggest that the coalescence time of CM/KP and XR populations was approximately 3.96 × 10^4^ generations ago (95% HPD: 0.50 × 10^4^–7.58 × 10^4^) (Table 3, Fig. 4). The average reproductive age of Korean pine is approximately 200 years^33^,^34^, which can be considered a reasonable estimate of Korean pine’s generation time. In this scenario, the coalescence time of the two regions was 7.92 million years ago (MYA), with the lower bound estimated as one MYA. Even using the initial reproductive age of Korean pine (80 years) as the generation time^35^, which usually underestimates the actual generation time, the coalescence time of CM/KP and XR populations was 3.17 MYA, with a lower bound estimated as 0.40 MYA, long before the last glaciation. Hence, it is certain that the two mountain regions sustained two separate populations for at least several glacial-interglacial cycles.

Although CM/KP and XR probably had separate refugia during the glaciation period, the populations from the two mountain ranges shared almost all dominant haplotypes in both nuclear and chloroplast markers (Fig. 1b and 2), which may be due to gene flow and ancestral polymorphism. It is possible that the CM/KP and XR refugia shared most genetic variation for several glacial cycles. However, we still observed new, unique mutants in each refugium. For example, haplotype H3 of *CL180Contig1_03* was unique to XR, and H4 of *0_1688_02* was unique to CM/KP (Fig. 2). From another perspective, homogenizing gene flow may obscure differentiation between the populations of the two mountain ranges. Our msABC simulation estimated that the number of migrants per generation from CM/KP to XR was slightly larger than one individual but that it was less than one individual in the reverse migration direction (Table 3, Fig. 4). Simplification of the ABC models results in a gene flow estimate that is a long-term average for the time since the separation of the two populations. When the two populations contracted during the LGM, gene flow might have been reduced due to population isolation. During the interglacial period, gene flow might have exceeded one individual (*N*_e_*m*) due to population expansion and the strong dispersal capability of pine pollen (discussed above). Wright^36^ suggested that when gene flow between populations exceeds one individual per generation, genetic differentiation will be eliminated.

The ABC simulation results suggested that the northern refugium located in XR would have been much smaller than the southern refugium in CM/KP. The effective population size of XR was less than 1200, nearly 50 times smaller than that of CM/KP (Table 3, Fig. 4). If both the CM/KP and XR regions sustained LGM refugia, the XR environment was probably harsher than that of CM/KP during the cold period. Scattered locations with suitable local environments (such as humid valleys) in XR might have provided habitats for relict populations in XR^21^. An ice cap was absent in NEA during the LGM, so the Korean pine might not be the only example of a species with northern microrefugia in the XR region. For example, a population of *Fraxinus mandschurica* is found in XR (near population TWH in our study, Fig. 1). This population possesses genetic variation that is divergent from other populations in NEA based on nuclear microsatellite data^15^. A population of *Quercus mongolica* near location FZ harbored a unique chlorotype with a very high frequency (Zeng *et al.* unpublished data), and this location has been considered a northern microrefugium. However, we again note that long-term average migration from XR to CM/KP is quite low, less than one individual per generation (*N*_e_*m*, Table 3, Fig. 4). We cannot exclude the possibility that gene flow during the interglacial period would be much larger, as discussed above. As the potential microrefugium in XR was located peripherally, it is possible that population expansion was constrained, after the LGM, compared to the southern macrorefugium in CM/KP. This is probably due to the suboptimal environment in XR and potential genetic constraints in small populations of refugia^7^. In addition, the flowering season of Korean pine is from mid-to late June^37^ and is under the influence of the summer monsoon in NEA. Hence, the northward monsoon may promote migration via pollen from CM/KP to XR while limiting the north-to-south pollen movement.

In summary, based on nuclear sequencing markers and ABC simulation statistics, we concluded that both the Changbai Mts. (including the Korean peninsula and the Sikhote-Alin Mts.) and the Xiaoxingan Range had relict populations of Korean pine during the LGM. The coalescence time between the populations of the two mountain ranges was at least 5000 generations ago, i.e., an estimated 0.4 million years ago. Although the two mountain ranges shared most of the total genetic variation, unique alleles exist in both regions for all studied markers. In contrast to the macrorefugia in the Changbai Mts. and its adjacent area, the microrefugium in the Xiaoxingan Range, combined with strong gene flow between regions, may have prevented genetic differentiation and eliminated the pattern of genetic variation over the species’ range.

Due to the limited samples from the Korean peninsula and the Sikhote-Alin Mts., we treated those two regions and the Changbai Mts. as a single unit. Although the samples from the Korean peninsula were limited, we found unique alleles occurring with low frequency there (Fig. 2). Some studies have reported that the Korean peninsula might be a refugium separate from the Changbai Mts^16^. Additional plant samples and details of refugia locations in the Changbai Mts. and the adjacent areas would provide more insight into the postglacial evolutionary history of Korean pine.

## Methods

### Sample Collection

During 2009–2012, we collected leaf samples from 75 adult trees in 21 natural populations covering the entire range of Korean pine in the NEA. From each population, 2–9 individuals were sampled, and the sampled individuals were at least 30 m apart from each other (Fig. 1, Table S1). The needles were dried and preserved in silica gel until DNA extraction.

### DNA extraction, amplification, and sequencing

Total DNA was extracted from needles using a Plant Genomic DNA Kit (Tiangen Biotech, Beijing, China). We selected ten polymorphic nsc loci based on 44 loci initially developed for other pine species^23^,^24^,^38^. These loci could be stably amplified with a single polymerase chain reaction (PCR) band via agarose gel electrophoresis. The primer sequences, annealing temperatures, size of each PCR product, and the putative function and structure of these loci are shown in Table 2. Three intergenic chloroplast fragments, *psb*A-*trn*H, *trnS*-*trnG* and *trnL*-*trnF*^39^, were examined in preliminary studies, and little genetic diversity was found in loci *trnS*-*trnG* and *trnL*-*trnF* (one of 100 individuals had a different haplotype at locus *trnS*-*trnG*). Therefore, we only report the results from *psb*A-*trn*H, which was polymorphic. PCR products were directly sequenced using an ABI 3730 automated sequencer (Applied Biosystems)from BGI Tech Co., Ltd., Beijing, China. Each haplotype found was also verified by a cloning method. A pGEM–T Easy Vector (Promega, Fitchburg,WI, USA) was used in the cloning procedure, and at least five clones were sequenced. All sequence data have been deposited in GenBank, with accession nos. KP182424-KP182921, KT993573-KT995096.

### Genetic diversity analysis

Sequences were aligned using the CodonCode Aligner 3.6.1 Program (http://www.codoncode.com/aligner/) with the “Muscle” module. Each individual sequence was independently checked by two researchers, and heterozygous sites were marked by hand. Sequences of all 10 nsc loci were phased in DNAsp 5.10.01 using default settings^40^. Among the 10 nsc loci, only *IFG8612* had two indels (3 bp and 55 bp). We replaced the two deletions of *IFG8612* with almost the same sequence as the inserts, with only one base pair difference. Hence, each indel was treated as a nucleotide substitution and assumed to evolve by nucleotide substitution.

Population genetic variation was assessed by the following parameters: the observed number of haplotypes (*H*), haplotype diversity (*Hd*), the number of segregating sites (*S*)^41^, nucleotide polymorphism at total sites (*θ*_*w*_), silent sites (*θ*_*ws*_), and the nucleotide diversity at total sites (*π*_t_), silent sites (*π*_s_), and nonsynonymous sites (*π*_a_)^42^. All of the loci were tested for departures from neutrality using Tajima’s *D*^43^, Fu and Li’s D* and F*^44^. The significance of each test was determined using 1000 coalescence simulations. The aforementioned statistics were computed using the DnaSP program v. 5.10.01^40^.

Median-joining networks were reconstructed in Network 4.6.1.2 for haplotypes found in each of the 10 nsc loci and the cpDNA^45^.

### Latitudinal gradient analysis of genetic diversity

Pairwise genetic differences within populations (*π*_Xi_) and between populations (*π*_XYi_) were calculated for each sampled population and each locus (i) using Alequin 3.5 software^46^. The total pairwise genetic differences over ten nsc loci within populations (*π*_X_) and between populations (*π*_XY_) were calculated by the sum *π*_Xi_ or *π*_XYi_ of each locus divided by the total sequence length of the ten nsc loci.

To verify the potential effect of sample size on genetic diversity within populations, rarefied allelic richness and private allelic richness were calculated using HP-Rare^47^,^48^. Haplotypes of each locus were coded as numbers and imported into the program. Only populations with 3 or more samples were used in this analysis. The two samples from population XUS were combined into BHS due to the approximate sample location. Six genes were set during the rarefied calculation.

The Pearson correlation coefficient between latitude and the genetic diversity indices was calculated and tested using R 3.1.0 (http://www.r-project.org/). Mantel tests of the correlation between genetic distance (*π*_XY_) and geographic distance (kilometers) were calculated using GenAIEx 6.1^49^.

### Historical demography analysis

To test the single refugium vs. multiple-refugia hypotheses for Korean pine, two ABC models were established. The single LGM refugium ABC model (SRM) hypothesized that CM/KP populations and XR populations coalesced after the LGM, while the multiple LGM refugia (MRM) ABC model hypothesized that CM/KP and XR populations coalesced before the LGM. In both models, the migration direction and magnitude were unrestricted. We had limited samples for KP and the Sikhote-Alin Mts. (three populations and 12 individuals total), so we treated populations in CM, KP and the Sikhote-Alin Mts. as a single southern population (marked as CM/KP), while we treated populations in XR as a northern population (marked as XR). Populations FZ, QS and RH were located far from the center of CM and near the XR region (Fig. 1). Hence, we did not include these three boundary populations in the ABC simulation. We performed the simulation with the program msABC^50^.

In the ABC models, we set the average mutation rate for the ten nsc loci as 1.31× 10^−9^ according to a previous estimate for *Pinus* species^51^. The initial effective population size was set as 10,000, which was close to the estimate based on the 10 loci. The generation time for Korean pine is unknown. Korean pine reaches maturity around 80–140 years^34^, whereas the data from a 5 ha clearcut plot of Korean pine in the NEA suggested that the average age of reproduction of Korean pine was approximately 200 years^33^. To avoid model selection bias due to the long generation time, we used a generation time of 100 years when we set the priors. In the SRM, we set the prior for the coalescence time of Korean pine as no more than the LGM (0–24,000 years before the present; the msABC-required transformation is 0–0.006). In the MRM, the coalescence time was set to be greater than the LGM. Korean pine is a Tertiary relict species^52^, so a large upper limit for the coalescence time was set as 10 million years before the present. Hence, the msABC-required prior transformation for the MRM was 0.0061–2.5. We set the northward and southward migration in both models as unlimited (4*N*_e_*m*: 0–1000). Details of the models and the msABC executable statements are shown in Fig. 3 and Table S2.

For each model, we simulated one million steps. Model selection was conducted in the statistics package abc in R 3.1.0. The “mnlogistic” method was used for model selection, with a tolerance rate of 0.001. We used 16 summary statistics that were closely related to gene flow and effective population size, including the mean and variance of segregating sites within populations and between populations *S*^41^, pairwise nucleotide differences within populations and between populations *π*^42^, genetic divergence (*F*_ST_) and shared allelic richness. The Bayes factor was used to determine the best supported model using the observed data^53^. To avoid random error, each model was simulated independently twice, using an independent model selection procedure.

For the best supported model, an additional four million steps were simulated. With the five million simulations, we estimated parameters such as the coalescence time of the CM/KP and XR populations, effective population size, and the migration rate between the CM/KP and XR populations. To ensure that the estimated range was located in the prior range, a log transformation was applied during the procedure. Neuronet’s method with nnet = 50 was used in the parameter estimation.

## Additional Information

**How to cite this article**: Bao, L. *et al.* Contributions of multiple refugia during the last glacial period to current mainland populations of Korean pine (*Pinus koraiensis*). *Sci. Rep.* **5**, 18608; doi: 10.1038/srep18608 (2015).

## Supplementary Material

Supplementary Information

## Figures and Tables

**Table 1:** Information about the ten nuclear single-copy loci used in this study.

Locus	PCR primers (5′–3′)	Annealing temperature (°C)	Alignment length (bp)	Putative function	Exon	Intron	Sources
*IFG8612*	F: TGTTAGCATGCAATCAATCAC	59	434	Late embryogenesis abundant protein	333–434	1–332	[1]
	R: CTGACAGAGTGGGCAGCTTCAT						
*a3ip2*	F: AATGCCAGGTTGGTGTTA	60	545	ABI3-interacting protein 2		1–545	[2]
	R: CAGCCTCAATTTGCTTTCC						
*erd3*	F: GAACGGGTCCGTACATTTTCTG	65	657	Early responsive to dehydration 3	1–165; 262–338; 484–657	166–261; 339–483	[2]
	R: TGCCAGATTGATTGGCATAGAA						
*nac1*	F: CTTCGGCTGTGGATGTATTGGA	63	738	Regulatory element	648–738	1–647	[3]
	R: ATCGTTTCAACTGCCTTGGCTT						
*gatabp1*	F: CGACCTTTGTGGAGGATTCTTTTATG	60	336	Regulatory element	7–288	1–6; 289–336	[3]
	R: CTTGGGCTTTCTTGCTATGGGTTTT						
*set-like-b*	F: TTATTTACATCCAACAGCGCATTT	63	489	Regulatory element	253–304; 482–489	1–252; 305–481	[3]
	R:GAAAGTATGGATTGCCAACTTGCAC						
*0_9389_01*	F: CAACTTCCCTCTCTTC	48	480	Nucleic acid binding protein	1–47, 264–328, 424–480	48–263, 329–423	[4]
	R: CATTGCCACAGTGGTTAC						
*CL180Contig1_03*	F: AAGTGAACAACGGCTG	57	579	Polygalacturonate 4-alpha-galacturonosyltransferase	1–31, 435–576	32–434	[4]
	R: TGGAGAAGGGAGAAGTG						
*0_1688_02*	F: GACTGACTATAACAGCC	54	628	Leucine-rich repeat family protein	1–154, 469–628	155–468	[4]
	R: CCCAAAATCACCAACAAC						
*0_12929_02*	F: GTTACAACATCAGCAATCAG	57	707	Protein kinase family protein	1–255, 606–707	256–605	[4]
	R: AAGCCAATCCACCAGTTATATACAG						

[1]^24^; [2]^25^; [3]^23^; [4]^38^.

**Table 2:** Nucleotide diversity of the ten nuclear single copy loci.

Locus	*S*	*H*	*π*_t_	*π*_s_	*π*_a_	*θ*_*w*_	*θ*_*ws*_	*Hd*	Tajima’s *D*	Fu and Li’s D*	Fu and Li’s F*
*IFG8612*	10	7	0.00329	0.00397	0.00018	0.00413	0.00453	0.478	−0.492	0.669	0.549
*a3ip2*	19	12	0.00866	0.00866	0.00000	0.00644	0.00644	0.816	0.959	−0.707	−0.088
*erd3*	5	4	0.00172	0.00239	0.00000	0.00136	0.00190	0.380	0.520	−1.223	−0.758
*nac1*	10	9	0.00254	0.00944	0.00072	0.00243	0.00698	0.411	0.112	−0.144	−0.063
*gatabp1*	13	8	0.00586	0.02030	0.00285	0.00693	0.01900	0.644	−0.395	−0.419	−0.490
*set*−*like*−*b*	4	5	0.00119	0.0000	0.00185	0.00149	0.00000	0.533	−0.385	−1.576	−1.401
*0_9389_01*	4	3	0.00260	0.00355	0.00000	0.00150	0.00205	0.419	1.356	−0.348	0.249
*CL180Contig1_03*	2	3	0.00023	0.00030	0.00000	0.00062	0.00081	0.131	−0.906	0.658	0.196
*0_1688_02*	7	5	0.00286	0.00461	0.00000	0.00200	0.00323	0.406	0.944	0.241	0.574
*0_12929_02*	8	7	0.00051	0.00045	0.00062	0.00203	0.00206	0.279	−1.710	0.377	−0.420
Average	8.2	6.3	0.00295	0.00537	0.00062	0.00289	0.00470	0.450	0.000	−0.247	−0.165

All summary statistic were calculated in DNAsp v. 5.10.01. For each locus, we calculated the observed number of haplotypes(*H*), haplotype diversity (*Hd*), the number of segregating sites (*S*)^41^, nucleotide polymorphism at total sites (*θ*_*w*_), silent sites (*θ*_*ws*_), and the nucleotide diversity at total sites (*π*_t_), silent sites (*π*_s_), and nonsynonymous sites (*π*_a_)^42^, Tajima’s *D*^43^, Fu and Li’s D* and F*^44^.

**Table 3:** Results of parameter estimation based on multiple LGM refugia ABC model in Fig. 3.

	*N*_S_	*N*_N_	*N*_a_	*T*	*M*_SN_	*M*_NS_
2.5% HPD	26854	719	9525	5032	0.16	0.06
Median	43413	869	53881	39568	1.08	0.24
Mean	43311	894	50573	39608	1.07	0.26
Mode	46494	830	63703	39044	1.28	0.24
97.5% HPD	62897	1107	85222	75836	2.08	0.55

*N*_N_ and *N*_S_ denoted the long-term effective population size of Changbai Mt. (including its adjacent regions) and Xiaoxingan Range, respectively. *N*_a_ was the effective population size of the most common recent ancestor of the two regional populations. *M*_SN_ and *M*_NS_ were the long-term average gene flow from Changbai Mt. to Xiaoxingan Range and from Xiaoxingan Range to Changbai Mt., respectively. *T* denoted the coalescent time of the two regional populations.
