# Supplementary material for: Contributions of multiple refugia during the last glacial period to current mainland populations of Korean pine (Pinus koraiensis)
Source: Sci Rep. 2015 Dec 22;5:18608. doi: 10.1038/srep18608 (PMC4686996; doi:10.1038/srep18608)
Supplement: Supplementary Information [file srep18608-s1.pdf]

Contributions of multiple refugia during the last glacial period to current mainland populations of Korean pine (*Pinus koraiensis*)

Lei Bao[1], Ayijiamali Kudureti[1], Weining Bai[1], Rongzhang Chen[1], Tianming Wang[1], Hongfang Wang[1,*], Jianping Ge[1]

[1] State Key Laboratory of Earth Surface Processes and Resource Ecology & College of Life Sciences, Beijing Normal University, Beijing, China

* Email: wanghf@bnu.edu.cn

* Telephone: 86-1058804806

Table S1 Geographic locations, located mountain, sample size(*N*) for the investigated populations of *Pinus koraiensis*.

Code	Longitude	Latitude	*N* [1]	Location	Country	Region [2]
BHS	126.99	37.65	2	Beihanshan	South Korea	KP
XUS	128.49	38.17	4	Xueyueshan	South Korea	KP
BS	124.80	40.78	3	Baishi	China	CM
RNZ	126.01	41.41	3	Renaozhen	China	CM
DS	125.00	41.93	3	Dasu	China	CM
CBS	128.07	42.06	3	Changbaishan	China	CM
LW	126.45	42.37	3	Longwan	China	CM
LHS	126.24	43.19	3	Lianhuashan	China	CM
WQ	130.18	43.35	3	Wangqing	China	CM
XBH	128.54	44.18	3	Xiaobeihu	China	CM
FZ	128.99	45.67	3	Fangzheng	China	CM*
QS	131.19	46.58	3	Qingshan	China	CM*
RH	133.58	46.83	9	Raohe	China	CM*
LE	131.50	43.18	6	Leopard park	Russia	CM
TU	136.55	45.06	6	Turney	Russia	Sikhote-alin Mt.
YL	129.38	46.63	3	Yilan	China	XR
JST	129.61	47.36	3	Jinshantun	China	XR
LN	130.43	48.06	3	Lingnan	China	XR
WY	129.19	48.21	3	Wuying	China	XR
TWH	129.87	48.59	3	Tangwanghe	China	XR
SS	126.78	49.48	3	Shengshan	China	XR

*:Not included in msABC analysis since these populations are the northern margin of Changbai Mt., close to Xiaoxingan Range.

[1]: Number of sampled individuals. The sample size should be 2*N* for diploid nuclear genome and *N* for haploid cpDNA.

[2]: CM, Changbai Mt.; KP, Korean peninsula; XR, Xiaoxingan Range.

Table S2 Command lines for the two demographic models used in the coalescent simulations conducted by msABC. File locfile.csv denotes the file ("locfile" as called in msABC manual) containing information about sample size of each locus, locus specific mutation rate (per locus per generation) and length of the locus (in base pair) for all 10 nuclear loci. File out.txt denotes the specific output file for summary statistics generated from every simulation for each demographic model.

Demographic model	msABC command
single LGM refugium	./msABC 120 1000000 --dur-mode -I 2 84 36 -m 1 2 -U 0 1000 -m 2 1 -U 0 1000 -en 0 1 -U 0.1 10 -en 0 2 -U 0.1 10 -ej -U 0 0.006 1 2 -eN 0 -U 0.1 10 --frag-begin --finp locfile.csv --N 10000 --frag-end --verbose -seeds 1 2 3 >out.txt
multiple LGM refugia	./msABC 120 1000000 --dur-mode -I 2 84 36 -m 1 2 -U 0 1000 -m 2 1 -U 0 1000 -en 0 1 -U 0.1 10 -en 0 2 -U 0.1 10 -ej -U 0.0061 2.5 1 2 -eN 0 -U 0.1 10 --frag-begin --finp locfile.csv --N 10000 --frag-end --verbose -seeds 105 5 30 >out.txt
